# Multilayered Polyelectrolyte Microcapsules: Interaction with the Enzyme Cytochrome *C* Oxidase

**DOI:** 10.1371/journal.pone.0112192

**Published:** 2014-11-05

**Authors:** Laura Pastorino, Elena Dellacasa, Mohamed R. Noor, Tewfik Soulimane, Paolo Bianchini, Francesca D'Autilia, Alexei Antipov, Alberto Diaspro, Syed A. M. Tofail, Carmelina Ruggiero

**Affiliations:** 1 Department of Informatics, Bioengineering, Robotics and Systems Engineering, University of Genova, Genova, Italy; 2 Department of Chemical and Environmental Sciences, University of Limerick, Limerick, Ireland; 3 Nanophysics, Istituto Italiano di Tecnologia, Genova, Italy; 4 PlasmaChem GmbH, Berlin, Germany; 5 Materials and Surface Science Institute, University of Limerick, Limerick, Ireland; Michigan State University, United States of America

## Abstract

Cell-sized polyelectrolyte capsules functionalized with a redox-driven proton pump protein were assembled for the first time. The interaction of polyelectrolyte microcapsules, fabricated by electrostatic layer-by-layer assembly, with cytochrome *c* oxidase molecules was investigated. We found that the cytochrome *c* oxidase retained its functionality, that the functionalized microcapsules interacting with cytochrome *c* oxidase were permeable and that the permeability characteristics of the microcapsule shell depend on the shell components. This work provides a significant input towards the fabrication of an integrated device made of biological components and based on specific biomolecular functions and properties.

## Introduction

Nanoengineered polyelectrolyte capsules (NPCs) are regarded as very promising nanosystems for a broad range of applications, including drug delivery, biosensors, bioreactors and artificial cells [1]–[3]. NPCs are fabricated with nanometer precision through layer-by-layer (LbL) assembly of a multilayered shell [4]. The process is based on the alternate adsorption of oppositely charged polyelectrolytes onto a sacrificial nano-/micro-particle core [5]–[7]. Once the multilayered shell is assembled, the core is dissolved by a complexing agent or in an acidic medium. The main advantages of this technique are its simplicity and versatility, since only very simple apparatus is required for NPC assembly with the ability to synthesize NPCs of different sizes and shapes dependent upon the building blocks. Moreover, it has been demonstrated that several functionalities in a particular NPC can be achieved by inclusion of different functional molecules, biomolecules, polymers and inorganic nanoparticles, both in the shell and in its hollow void [8]. Finally, various stimuli-responsive NPCs have been developed, which are able to vary the permeability of their shell for encapsulation and release after a specific stimulus such as pH, laser irradiation, ultrasound, magnetic fields and enzymatic digestion [9]–[13].

In this context, the inclusion of active functional proteins in nano-/micro-sized systems is of particular interest in the biomedical field. Such functional systems can be applied in biocatalysis to perform specific reactions in restricted volumes, in diagnostic and therapeutic interventions, monitoring *in vivo* parameters and releasing cargo molecules under specific conditions, and in functional studies of important classes of proteins [14]–[17]. Membrane proteins are one of the most important classes of proteins since they mediate the interaction between the biotic cellular environment and the abiotic external medium. They are responsible for various biological processes, such as cell signaling-transduction pathways and bioenergetics. Therefore, membrane proteins are of great importance for research and healthcare applications.

Herein the functionalization of NPC shells by a membrane protein, cytochrome *c* oxidase is described. Cytochrome *c* oxidase (Cyt*c*O) is the terminal enzyme in the respiratory chain and is located in the inner mitochondrial or bacterial membrane. Cyt*c*O catalyzes the reduction of molecular oxygen to water through electrons delivered by cytochrome *c*. The reaction is linked to the pumping of one proton across the membrane for each electron transferred from its substrate cytochrome *c* to O_2_, although this ratio is reduced in certain types of Cyt*c*O. The resulting proton gradient activates ATP synthase to generate ATP from ADP. This chemical process represents one of the most critical aspects of cellular respiration. The reconstitution of energy transducing proteins, such as cytochrome *c* oxidase, into biomimetic membranes has been proposed for the fabrication of active materials with broad applicability [18], [19]. To our knowledge, only one paper deals with the functionalization of polyelectrolyte multilayers with a membrane protein, namely bacteriorhodopsin [19].

In our work, Cyt*c*O, specifically the *ba*_3_-oxidase from *Thermus thermophilus* [20], was incorporated by electrostatic interactions into the shell of NPCs. Dissipative quartz crystal microbalance (QCM-D) was used to monitor the step-by-step shell assembly and to evaluate its thickness and the mass of immobilized Cyt*c*O. The residual catalytic activity of the immobilized Cyt*c*O was determined by monitoring the oxygen consumption in the presence of reduced cytochrome *c*_552_. The influence of Cyt*c*O on the permeability properties of NPCs was evaluated by confocal laser scanning microscopy. In order to explore and characterize the mechanisms governing the permeability variation induced by Cyt*c*O, both synthetic NPCs, made from polystyrene sulfonate and polyallylamine, and biocompatible and biodegradable NPCs, from the polysaccharides chitosan and pectin, were studied in the presence of Cyt*c*O.

The results reported here could be useful both for functional studies on the interaction of NPCs with intracellular enzymes and for applications in drug delivery, biosensing and protein driven current production.

## Materials and Methods

### Materials

All reagents were obtained from Sigma-Aldrich. Cationic poly (allylamine hydrochloride) (PAH, Mw 70 kDa), anionic poly (styrene sulfonate) (PSS, Mw 70 kDa), chitosan (medium Mw) and pectin (high degree of methylation 70–75%) were used as polyelectrolytes for NPCs assembly.

Calcium chloride, sodium carbonate and ethylenediaminetetraacetic acid (EDTA) were used for calcium carbonate microparticle synthesis and subsequent dissolution.

Potassium chloride, Tris-HCl buffer, sodium hydroxide, dodecyl-β-D-maltoside, HEPES, ascorbate and tetramethylphenylene-diamine (TMPD) were used for Cyt*c*O catalytic activity evaluation.

The water used in the experiments for the preparation of solutions was purified by Milli-Q system and had a resistance of 18.2 MΩ cm.

The permeability of the capsules was examined with confocal microscopy by adding into the sample 5 µl of Rhodamine B (Sigma-Aldrich) at a concentration of 1 mg/ml in Milli-Q water.

### Purification of cytochrome *ba*_3_-oxidase and cytochrome *c*_552_

The cytochrome *ba*_3_-oxidase was purified essentially as previously described [21], [22]. *T. thermophilus* HB8 cells were fermented in a bioreactor under a reduced oxygen tension (0.05 volume of air per volume of medium per minute) for a maximal *ba*_3_ Cyt*c*O expression at 70°C. Frozen cells (100 g) were resuspended in 500 mL of lysis buffer (0.1 M Tris-HCl pH 7.6, 100 mM NaCl). After achieving homogeneous resuspension, 400 mg of lysozyme was added and left at room temperature (ca. 20°C) for 4 h under stirring prior to centrifugation (20,000 *g*, 1 h, 4°C). The supernatant containing cytochrome *c*_552_ was discarded (in contrast to the previous protocol) as a recombinant expression system has been established instead. The pellet, containing membrane proteins, was resuspended in the lysis buffer and centrifuged again; this step was repeated once. The membrane was solubilized in 500 mL of lysis buffer with the addition of 5–6% Triton X-100 and stirred overnight at 4°C. Insolubilized materials were removed by a centrifugation step as before. The supernatant, diluted to 5 L of H_2_O, was loaded onto a 150-ml glass column (XK 26) packed with Q Sepharose FF (GE Healthcare) equilibrated with 10 mM Tris-HCl pH 7.6, 0.1% Triton X-100. Fractions containing *ba*_3_ Cyt*c*O, eluted with about 50–100 mM NaCl, were pooled and dialyzed in an 8-kDa cutoff membrane against 10 mM Tris-HCl pH 7.6, 0.1% Triton X-100. The bath volume was ten times the total fractions volume. The protein was then loaded onto EMD TMAE-650 (Merck) in an XK26 column, and the Triton detergent exchanged to 0.025% DDM. The *ba*_3_ Cyt*c*O was eluted with NaCl, concentrated and injected onto a Superdex 200 gel filtration column equilibrated with 50 mM Tris-HCl pH 7.6, 0.025% DDM. Peak fractions, eluted at ca. 60 mL, were pooled and concentrated to 20 mg/mL prior to snap-freezing with liquid nitrogen and storage at −80°C.

The cytochrome *c*_552_ purification was performed according to previously published procedure [23], except that the gene for cytochrome *c*_552_ (TTHA1423; GenBank ID: 3169955) was amplified and cloned into pET-22b(+) at NcoI/XhoI sites without any affinity tags. The *E. coli* cells (30 g from 10 L expression in LB broth) were lysed and the periplasmic fraction was dialyzed against 10 mM Tris-HCl pH 7.6. Protein purification was performed on CM Sepharose and EMD COO^−^ columns before a gel filtration step on Superdex 75, as described previously for native cytochrome *c*_552_ [24].

### NPCs preparation

The NPCs were assembled onto positively charged calcium carbonate sacrificial microparticles (6 µm in diameter), obtained by mixing calcium chloride and sodium carbonate solutions according to the reaction [25], [26]:
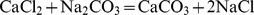



The NPCs were fabricated according to a well-established procedure, as previously described [5]. Briefly, 10^8^ CaCO_3_ particles were covered by successively deposited layers of anionic and cationic polyelectrolytes. Specifically, PSS and PAH were prepared in pure water at concentration 2 mg/ml, pH 6.5 and their adsorption time was 10 min. The polysaccharides chitosan and pectin were used at concentration 0.5 mg/ml. Chitosan was dissolved in 0.1 M acetic acid, while pectin was dissolved in pure water; their pH was then adjusted to pH 5 with 0.1 M NaOH and their adsorption time was 20 min. Four bilayers were deposited onto the surface of the particles; after each deposition step, the dispersion of covered particles was centrifuged (2500 rpm for 5 min) and the precipitated covered particles were separated from the solution. These particles were washed three times in pure water, in the case of PSS and PAH, and in water at pH 5, in the case of chitosan and pectin, with successive centrifugation and separation steps.

Particles were then dissolved by their dispersion in 0.5 M EDTA pH 5 followed by three washings in pure water. Finally, NPCs were dispersed in 20 mM Tris-HCl pH 8.5 for their subsequent functionalization with Cyt*c*O. Cyt*c*O at a concentration of 0.025 mg/ml (in 20 mM Tris-HCl pH 8.5) and 0.05% dodecyl-β-D-maltoside was added to the hollow NPCs and allowed to adsorb for 30 min. This step was followed by three washings in 20 mM Tris-HCl pH 8.5.

### Quartz Crystal Microbalance

The shell growth process was performed using both a QCM working in air and a QCM-D. In the first case, we used a gauge developed for this purpose by quartz crystal oscillators with a resonance frequency of 10 MHz and stability better than 1 Hz [27]. Quartz crystals were washed with acetone, dried under a nitrogen flux and then used for the deposition of the multilayer. A layer of PAH was first deposited in order to impart a positive charge to the surface for the following deposition of the structure (PSS/PAH)_4_/Cyt*c*O at pH 8.5. Since the quartz crystal surface is mostly negatively charged, PAH was deposited as the first layer. The multilayers (PAH/PSS)_4_/Cyt*c*O and (PAH/PSS)_4_/PAH/Cyt*c*O were characterized to investigate the degree of interaction of *ba*_3_ Cyt*c*O molecules with a terminal positive and negative layer.

Each layer was deposited on both sides of the resonator, washed and afterwards dried under a nitrogen flux. The change in resonance frequency was measured after each assembly step and correlated to the adsorbed mass (Δm, ng) by the Sauerbrey equation [28].
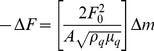
(1)where F_0_ is the resonance frequency of the quartz crystal oscillator, A is the area of the electrode, ρ_q_ is the quartz density, and µq is its shear modulus.

The following equation was derived from (1) and used in the present work:
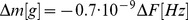
(2)


QCM-D was used to further characterize the multilayers. The QCM-Z500 instrument (KSV Instruments, Helsinki, Finland) measuring principle is based on the analysis of the quartz crystal impedance at multiple overtones [29]. An equivalent circuit model was fitted with the impedance curve and the obtained parameters were used to calculate the mechanical properties of the added layers such as mass, density and thickness [30], [31]. PSS/PAH multilayers were deposited on gold-coated 5 MHz AT-cut quartz crystals. Before adsorption, the quartz crystals were cleaned with H_2_SO_4_ at 150°C for 20 min followed by washing in pure water. A Teflon liquid chamber with a volume of 2 ml was used in the experiments. The solutions used for the assembly and washing steps were alternatively introduced into the measurement chamber, and were left in contact with the quartz crystal for 10 min for polyelectrolyte deposition. After each adsorption step, pure water was poured into the chamber and left in contact with the crystal for 1 min in order to remove the unabsorbed molecules. The data analysis was performed using the QCM Impedance Analysis software (KSV Instruments, version 3.11).

### Measurements of Ζ-potential

Laser Doppler Microelectrophoresis for ζ-potential measurements were done with the Malvern Zetasizer Nano ZS (Malvern Instruments) at 25°C. Measurement of ζ-potential was done in folded capillary cells with Tris HCl buffer 20 mM at pH 8.5 and 7.4 as dispersant, calculated using the Henry equation with Smoluchowski approximation.

### Measurements of Cyt*c*O activity

The catalytic activity of Cyt*c*O was investigated polarographically with recombinant *T. thermophilus* cytochrome *c*_552_ produced in *E. coli* [32]. The assays were performed at 25°C in a 5 ml vessel with a Clark electrode combined with an oxygen measuring unit (Oxygraph, Rank Brothers Ltd., UK) under continuous stirring. The reaction buffer was 20 mM Tris-HCl pH 7.4, 0.05% dodecyl-β-D-maltoside and 10 mM ascorbate (total volume 4.7 ml). The activity of Cyt*c*O was determined in the presence of 1 mM TMPD and reduced cytochrome *c*_552_ at a concentration of 28 nM.

The TMPD auto-oxidative oxygen consumption rates were always determined prior to enzyme addition in order to obtain true enzyme-catalyzed values. Desalting of ascorbate-reduced cytochrome *c*_552_ was achieved by a Sephadex G-25 gel filtration column.

### Confocal Laser Scanning Microscopy

Confocal laser scanning microscopy (CLSM) was performed to study the permeability variation looking mainly at the entrance of the dye molecules (rhodamine) from the environmental solution into the capsules volume. The images were obtained using a Leica TCS SP5 STED-CW (Leica Microsystems, Mannheim, Germany) inverted confocal laser scanning microscope equipped with a super-continuum laser covering the visible spectrum in the range between 470 and 640 nm. The images were collected using a Leica 100× HCX PL APO STEDorange NA 1.40 oil immersion objective (Leica Microsystems CMS, Mannheim, Germany) with an excitation at 561 nm and an emission between 570 and 620 nm, with no lines averaging at a speed of 1000 Hz per line, a pixel dwell time of 2 µs and a pinhole size of 0.8 Airy. Under this imaging configuration, typical confocal resolution is of the order of 200 nm in the lateral and 500 nm in the axial direction.

## Results and Discussion

The aim of our work was to fabricate and characterize NPCs functionalized with the enzyme Cyt*c*O. For this purpose, the *ba*_3_-oxidase from *T. thermophilus*, an extremely thermophilic bacterium, was immobilized on the outer surface of LbL assembled NPCs. It was previously demonstrated by Ladam and co-workers [33] that proteins strongly interact with polyelectrolyte films whatever the sign of the charge of both the multilayer and the protein. When the charges of the multilayer and the protein are similar, one usually observes the formation of dense protein monolayers. It was demonstrated also that, when the protein and the multilayer become oppositely charged, the adsorbed amounts of protein are usually larger, leading to the formation of protein layers extending up to several times the largest dimension of the protein [33]. In our work, the 2D LbL assembly of the multilayered shell was first characterized step-by-step by QCM. Specifically, synthetic anionic PSS and cationic PAH were used for the formation of the multilayer. (PSS/PAH)-NPCs were chosen as the most studied LbL NPCs system, which is fully characterized in terms of stability and permeability. Firstly, the interaction of Cyt*c*O with multilayers possessing a negative or positive terminal layer was investigated by the QCM working in air. For both multilayers, the frequency shift due to the deposition of each successive layer of polyelectrolyte onto the quartz crystal showed a gradual growth of the multilayer as a function of the assembly cycles. Comparing the results obtained for the two structures, the multilayer with a positive PAH terminal layer was found to be the better one as relates to Cyt*c*O molecules surface density. Specifically, the estimated surface densities for Cyt*c*O molecules were found to be 273±11 ng/cm^2^ and 129±5 ng/cm^2^ for positive and negative terminal layers respectively. This can be explained considering that the isoelectric point (pI) of *ba*_3_-oxidase is 7.3, and at the working pH of 8.5 the total surface charge of *ba*_3_-oxidase molecules is mainly negative, resulting in a stronger interaction with PAH terminated multilayer.

**Figure 1:**
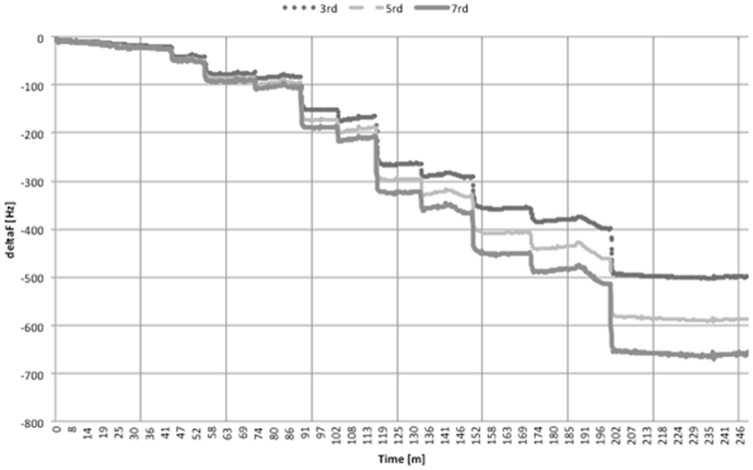
QCM-D result showing the in situ build-up of the multilayer PAH-(PSS/PAH)_4_/Cyt*c*O films at a pH 8.5. Frequency changes are recorded as a function of time for the 3^rd^, 5^th^ and 7^th^ armonics.

The assembly of the multilayer PAH-(PSS/PAH)_4_/Cyt*c*O at pH 8.5 was further characterized in real-time and in liquid environment by QCM-D. The *in-situ* QCM-D analysis of the formation of multilayers provides the total mass, polymer and water, adsorbed onto the quartz crystal surface at each deposition step. Highly hydrated multilayers tend to behave as soft viscoelastic hydrogels rather than rigid films. The loss of rigidity is detected by QCM-D. For rigid films, the frequency changes upon increasing film mass are the same for all overtones. For viscoelastic gels, Δf versus time curves for different overtones do not superimpose. This feature is apparent in kinetic QCM-D traces recorded during the buildup of PAH-(PSS/PAH)_4_/Cyt*c*O multilayers after the third deposited layer (Figure 1).

**Figure 2:**
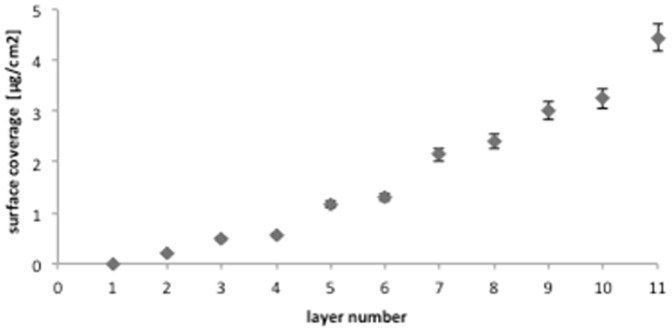
Surface coverage grow-up of the multilayer (PAH/PSS)_4_/PAH/Cyt*c*O at pH 8.5 as a function of the number of layers, calculated with the Voigt model by QCM-D. Step number 11 correspond to surface coverage value of the multilayer after the addition of Cyt*c*O.

For this reason, the data were analyzed using the Voigt-based model. In this model, the adsorbed film is represented by a single Voigt element consisting of a parallel combination of a spring and dashpot to represent the elastic (storage) and inelastic (damping) behavior of a material, respectively. The evolution of the surface coverage recorded during the deposition of the multilayer PAH-(PSS/PAH)_4_/Cyt*c*O is presented in Figure 2. The total thickness of the hydrated multilayer, constituting the NPC shell, was evaluated to be 45±3 nm, including the Cyt*c*O layer having a thickness of about 11 nm.

At this point, NPCs were prepared by deposition of the multilayer (PSS/PAH)_4_ onto CaCO_3_ micro-particles as previously described [5]. The micro-particles were then dissolved by complexation with EDTA to obtain hollow NPCs. The dissolution process was followed through optical microscopy. The obtained NPCs were then functionalized with Cyt*c*O.

The surface charge of functionalized and plain (PSS/PAH)_4_ NPCs was followed by microelectrophoresis. At the assembly pH 8.5 the surface potential of plain (PSS/PAH)_4_ NPCs was found to be +12 mV. Cyt*c*O was then adsorbed and the surface potential decreased to -2 mV, indicating that the deposition of Cyt*c*O was successful. The surface potential was also checked at the activity test pH 7.4, which is very close to the pI of ba3 Cyt*c*O (7.3). This characterization was carried out in order to verify that under activity test conditions Cyt*c*O molecules where still adsorbed onto the NPCs surface. At pH 7.4, the surface potential of plain (PSS/PAH)_4_ NPCs was found to be increased to +28 mV. This increase can be explained taking into account that the pI of PAH is 9. In the case of Cyt*c*O functionalized (PSS/PAH)_4_ NPCs, the surface potential decresead to +8 mV. The difference between the surface potentials of the two samples indicates that Cyt*c*O molecules are still adsorbed onto the surface of the NPCs. This behaviour at a pH close to the pI of LbL assembled proteins has been already reported in the literature. The LbL self-assembly process is primarily govened by electrostatic interactions. However it was demonstrated that hydrogen bonding, hydrophobic and other types of interactions are also involved [34]–[36]. For example, Caruso and co-workers [37] successfully immobilized by LbL IgG molecules on the outer surface of a polyelectrolyte multilayers at a pH close to the IgG pI, which indicates the importance of hydrophobic interactions.

**Figure 3:**
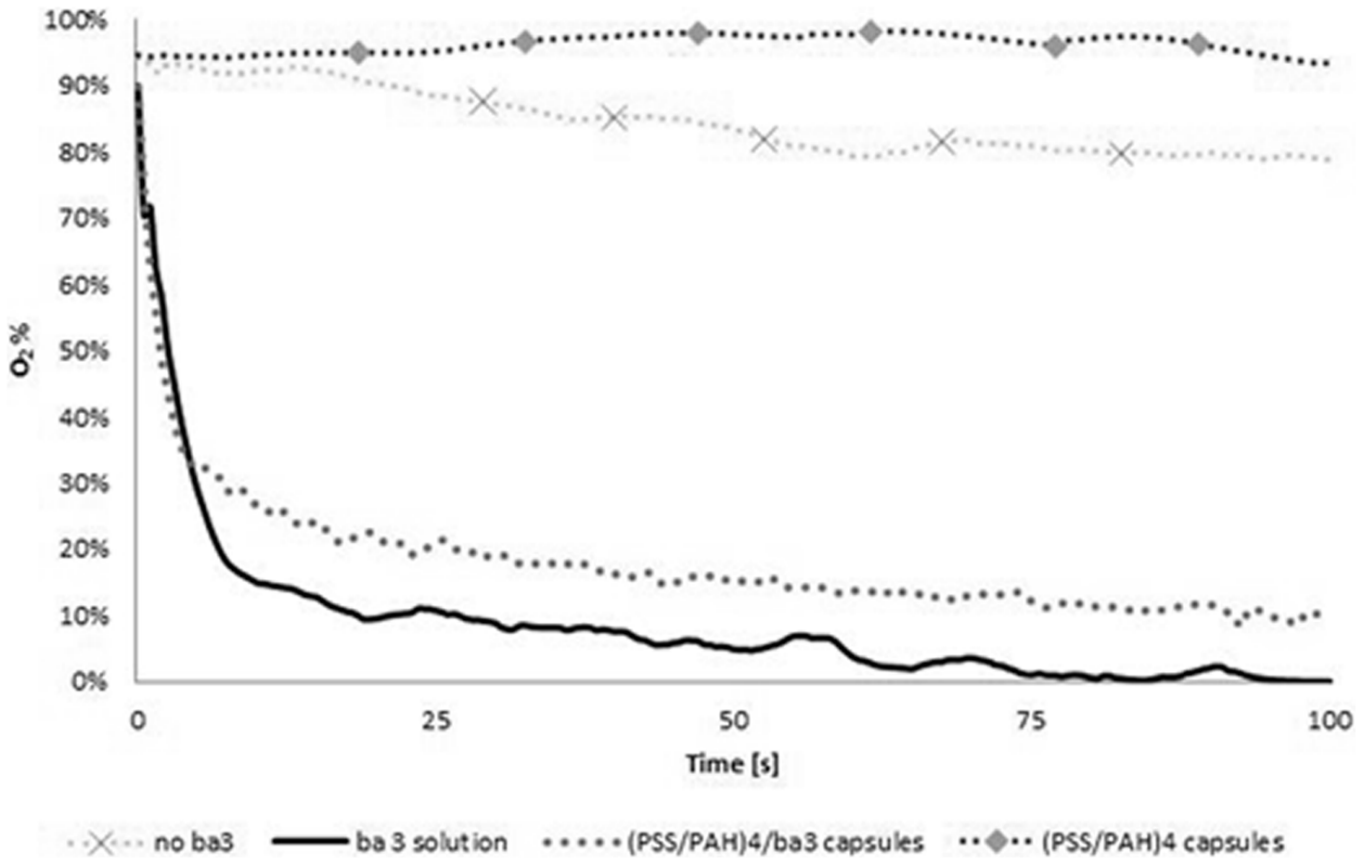
Cyt*c*O redox activity measured polarographically. The O_2_-consumption as a function of time for reaction mixture without Cyt*c*O, Cyt*c*O in solution, PSS/PAH)_4_/Cyt*c*O NPCs and PSS/PAH)_4_ NPCs. 100% indicate the initial amount of O_2_ in all the different samples.

The effective functionalization of the NPCs with Cyt*c*O molecules and their residual catalytic activity were then assessed polarographically by measuring the O_2_-consumption rate accompanied to the oxidation reaction of reduced cytochrome *c*_552_. The O_2_ consumption as a function of time for the functionalized and plain (PSS/PAH)_4_ NPCs was determined and compared with that of Cyt*c*O molecules in solution and for the reaction mixture without the addition of Cyt*c*O (Figure 3).

The QCM data on the surface density for the adsorbed Cyt*c*O molecules were used to estimate the mass of Cyt*c*O deposited onto the surface of the spherical NPCs. The results showed, as could be expected, that the O_2_ consumption in presence of reduced cytochrome *c*_552_ for plain NPCs and that for the reaction mixture without Cyt*c*O were very slow and not significant. On the contrary, it is interesting to note that in the case of both functionalized NPCs and Cyt*c*O molecules in solution, the addition of reduced cytochrome *c*_552_ immediately activated the redox reaction, resulting in O_2_ consumption, which was very fast in the first few seconds of the experiment, and after about 100 s, the solution was practically anaerobic. The activity of Cyt*c*O functionalized NPCs, in terms of percentage values of O_2_ consumption, was found to be comparable to the activity of the same concentration of free Cyt*c*O in solution. It might have been expected that Cyt*c*O activity is intervened by incorporation into NPCs because it has been found that charge-charge interactions and enzyme entangling can prevent substrates from reaching the active site [38].

**Figure 4:**
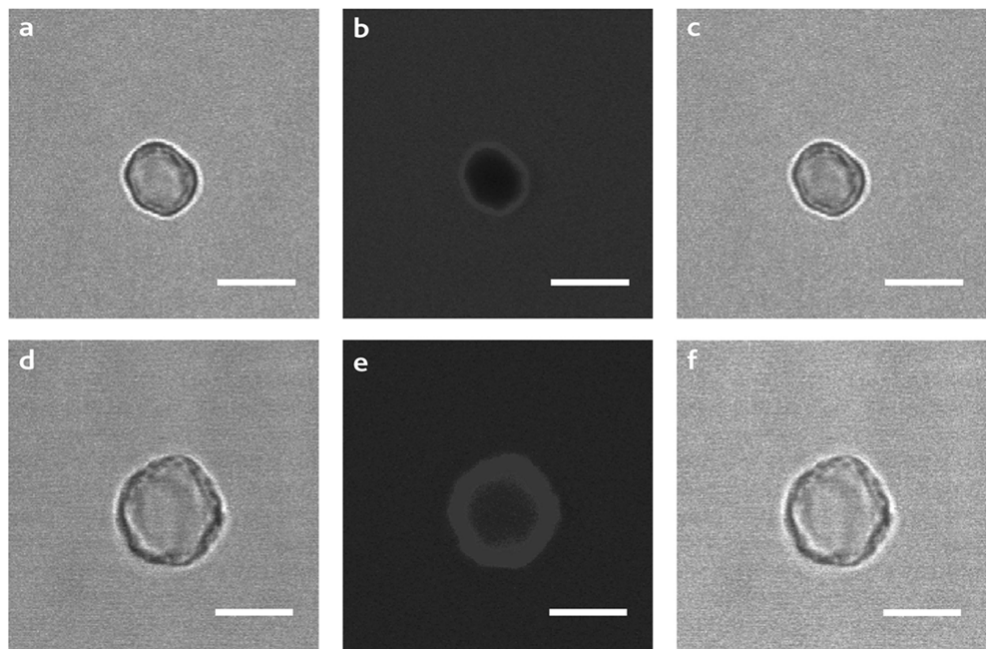
Confocal images of NPCs in the presence of rhodamine. Forward (a, d), confocal (b, e) and merged (c, f) images of empty capsules (top) can be compared with the corresponding images of (PSS/PAH)_4_/Cyt*c*O NPCs (bottom). The scale bar is equivalent to 5 µm.

Finally, the functionalized capsules were imaged by CLSM in order to characterize their permeability properties. Namely, the (PSS/PAH)_4_ and (PSS/PAH)_4_/Cyt*c*O NPCs were imaged in 20 mM Tris-HCl pH 8.5 in the presence of rhodamine. As expected, the (PSS/PAH)_4_ multilayered shell was found to be without defects and thus impermeable to the dye molecules, whereas the (PSS/PAH)_4_/Cyt*c*O multilayered shell was permeable (Figure 4).

**Figure 5:**
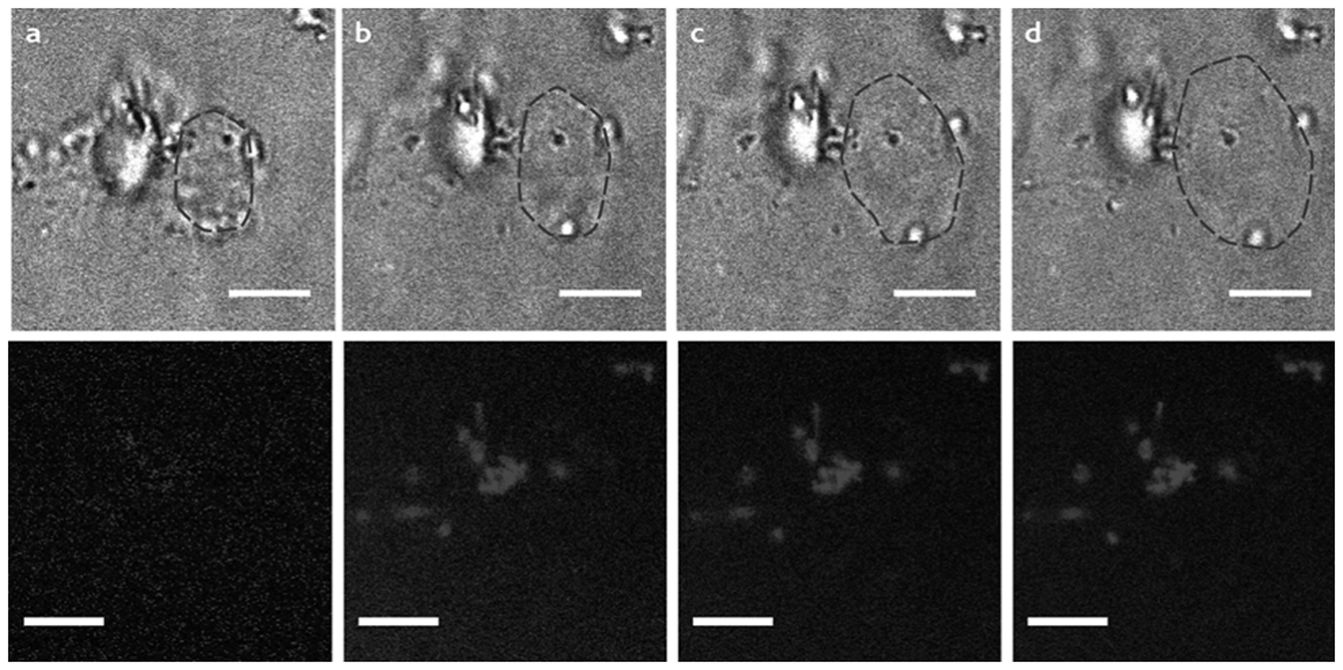
(CHI/PEC)_4_/Cyt*c*O NPCs sequence of images before (Panel A) and after addition of dye (B–D). The dashed line indicates the border of one (CHI/PEC)_4_/Cyt*c*O NPCs which upon addition of the dye swells and finally explodes. Scale bar is 5 µm.

In another experiment, NPCs were prepared using two weak polyelectrolytes. The cationic chitosan and anionic pectin were used for this. Specifically, we used a form of pectin with a high degree of methylation (70–75%), which is inversely proportional to the pectin charge density. Charge density plays a pivotal role in determining the stability of the multilayer [39]. The formation of stable films is not possible below a minimum charge density. Therefore, we can postulate that in the chitosan/pectin multilayer, there is an excess of positive charge and thus multilayer results in a more unstable and loose structure. Taking into consideration that the p*K*_a_ of chitosan and pectin are ca. 6.8 and 3.6, respectively, the NPCs were assembled at pH 5 to achieve a good degree of ionization for both polysaccharides, and then functionalized with Cyt*c*O. The NPCs morphology was checked by optical microscopy during all the preparation steps. It was observed that after Cyt*c*O functionalization and related washing steps, the majority of the capsules were destroyed or deformed. The functionalized capsules were then imaged by CLSM. It was observed that when the surrounding environment was perturbated by the addition of dye, the capsules suddenly “exploded” (Figure 5). On the bases of these observations, it can be hypothesized that Cyt*c*O molecules strongly interact with the multilayer and that different interaction types are involved, such as hydrophobic ones This inteaction leads to the formation of defects, which determine an increased permeability, for strongly bound multilayers (PSS/PAH), and to a loose and instable structure for weakly bound multilayers (chitosan/pectin).

Moreover, it has been demonstrated that the interfaces between layers in polyion films are not sharp and partial interpenetration (30–40% of their thickness) between neighbor layers takes place [40], [41], [33]. We can then hypothesize that Cyt*c*O molecules are somehow entangled in the polyelectrolyte multilayers.

The integration of Cyt*c*O within multilayered polyelectrolytes structures offers the possibility of tuning the properties of the capsule shell, designing the shell composition and thus the overall structure. Moreover, it can be hypothesized that by varying the number of Cyt*c*O molecules, adsorbed onto the outer surface of the NPCs, it would be possible to modulate their interaction with the shell and thus to control its permeability.

## Conclusions

In conclusion, the obtained results show that the external surface of polyelectrolyte capsules can be functionalized with redox-driven proton pump Cyt*c*O molecules in a controlled way. The immobilized enzyme remained active with respect to the catalysis of O_2_ reduction to H_2_O. Finally, it was observed that the permeability properties of the NPCs, having different composition, are strongly affected from the interaction with Cyt*c*O molecules. The results demonstrate that the NPC permeability is affected by the presence of membrane-integral enzymes depending on its composition. Therefore, the interaction of NPCs with Cy*c*O can be regarded as an *in vitro* characterization of *in vivo* properties of such systems. Considering the flexibility of the LbL method, a range of membrane proteins can indeed be incorporated into the capsule structure. In the present work, Cyt*c*O was chosen as a proof-of-concept, extendable to other proteins. Functional nanostructured colloidal systems are highly interesting both for the functional studies of energy transducing proteins, such Cyt*c*O, and for other biological-based applications including cellular compartment-targeted drug delivery, biosensing and biofuel cells.
